# Method for Nest Detection of the Yellow-Legged Hornet in High Density Areas

**DOI:** 10.3389/finsc.2022.851010

**Published:** 2022-02-28

**Authors:** Sandra V. Rojas-Nossa, Patricia Álvarez, Josefina Garrido, María Calviño-Cancela

**Affiliations:** Department of Ecology and Animal Biology, Faculty of Sciences, University of Vigo, Vigo, Spain

**Keywords:** colony, control, detection, invasive hymenopterans, nest, *Vespa velutina*

## Abstract

The Asian hornet *Vespa velutina* is a social predator that has invaded several countries of Europe and Asia, impacting pollinators, apiculture and human health. One of the few effective control methods developed so far is the early destruction of nests. However, they are often built within dense vegetation, being difficult to detect. The aim of the method described here is to detect nests with a simple procedure, utilizing readily available materials, for widespread use in infested areas. The method has two phases, the first phase involves capturing and marking hornets, lured to a protein bait, and recording the flight directions of individuals to the nest and the time needed to complete a bait-nest-bait round trip, to estimate the distance. Collecting this information from two (or more) bait stations allows to delineate the approximate location of the nest. The second phase aims to determine the precise location of the nest, using sugary baits in the vicinity of the nest and conspicuous marks attached to the released hornets, to visually follow them up to their nest. This method is an alternative to other methods that are either ineffective in areas with high nest density or require expensive equipment and specialized training.

## Introduction

The Yellow-legged hornet or Asian hornet (*Vespa velutina* Lepeletier, 1,836) is an invasive species with detrimental impacts on biodiversity, human health and economic sectors (1). This eusocial vespid is a ferocious predator of insects, especially pollinators, which leads to impacts to the pollination services of native plants (2). Honeybees are often their most preferred prey, affecting apiculture (3, 4).

Early detection and destruction of colonies before new generations of reproductive individuals emerge is to date the most effective strategy to control the invasive population of *V. velutina* (5). Colony nests are built of wood fibers chewed and mixed with saliva (6) in a variety of artificial and natural substrates, often hidden among the vegetation, or high in the tree canopy. This hinders early detection, since nests are difficult to distinguish from leaves and branches. As a result, most nests are only found when leaves fall from the trees in autumn, just before the collapse of the colony and thus too late to preclude the emergence of the next sexual generation. Developing practical and inexpensive procedures for early detection of nests is therefore crucial for effective population control (7).

Several methods have been used for nest detection. In Asia, the visual tracking of *Vespa* workers is a traditional method used to find nests (8), with hornets being attracted to a living bait and marked with a feather or bamboo piece attached to the hornet petiole to follow them to the nest. Another approach is to find foraging routes from carbohydrate-based bait stations, marking individuals, recording return times and moving stations along the flight path to get closer to the nest (9). In the latter stages, conspicuously marked hornets may be followed visually directly to the nest. Leza et al. (10) used also a visual method based on recording vanishing directions of flying hornets from bait stations and triangulation. These methods are successful in areas with low density of nests. However, in highly infested areas, baits are visited by hornets from different nests, making the tracing of routes confusing. The use of specialized techniques such as radio-tracking (5), harmonic radar (11, 12) or thermal imaging (13) can assist with minimizing these problems, but require considerable investment in equipment and specialized training, and may require additional licenses to operate according to regulations in some countries. In this contribution, we describe an alternative method of visual tracking that is simple to execute and has proven to be effective in areas with a high density of nests and dense vegetation.

Our observations in the field of the foraging behavior of *V. velutina* showed that, when workers find a carcass, a common protein source to feed the brood (6), they make several flights taking pellets of food to the nest before returning again, with a high fidelity in the routes followed and the time required for the round trip. The method described hereafter makes use of this scavenging behavior of *V. velutina* to locate their nests by using a combination of triangulation of flight directions and estimation of nest distance to define the proximate nest locations. This can be followed by additional visual tracking of individuals from carbohydrate-based baits set in this proximate area, to determine the precise nest location if not already located during phase one.

## Materials and Methods

The method was developed in a southern coastal area of the province of Pontevedra, Galicia, in NW Spain, in rural areas with patchy landscapes, with a mixture of houses, gardens, orchards, tree plantations mainly of eucalypts (*Eucalyptus globulus*), and pines (*Pinus pinaster*), shrublands dominated mainly by gorse (*Ulex* spp.), heath (*Erica* spp.), broom (*Cytisus striatus*) and blackberries (*Rubus ulmifolius*), and small patches of native forests (with *Quercus robur, Salix atrocinerea, Castanea sativa*, and *Laurus nobilis*, among others). *Vespa velutina* was recorded for the first time in this area in 2012 and the population has reached high abundances since then, with approx. 13,000 nests being detected and destroyed by the local administration during 2020 in 285 municipalities of the province (Xunta de Galicia Unpublished data, 2020). A total of 13 nests in three different areas of Pontevedra and two seasons (2017 and 2021) were used to develop and test the method described.

### Method Description

**Figure 1:**
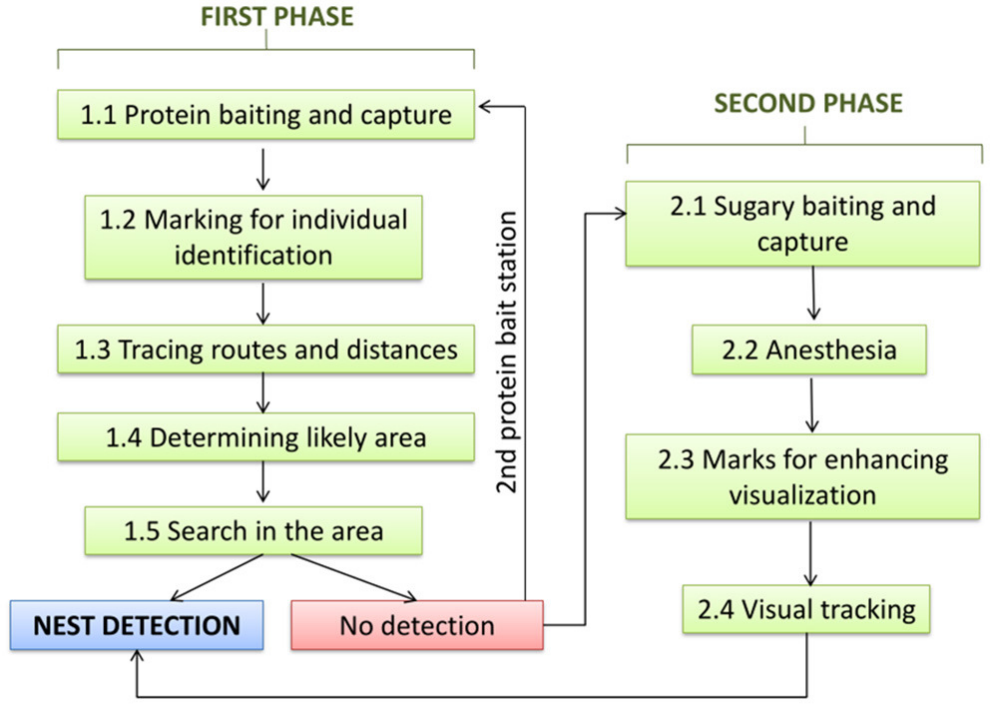
Protocol for the detection of nests of the Yellow-legged hornet: Phases and steps.

The method can be divided in two phases (Figure 1), with a first phase based on protein baits at which hornets are captured and marked with color codes on the thorax and/or abdomen, so that they can be identified individually. Fresh skinless fish or chicken (10) worked well as protein baits, although shellfish or shellfish scraps can also be used. Placing the baits in areas without visual obstacles and at eye level facilitates the monitoring of individuals when leaving the bait toward their nests. For marking, color paints used by beekeepers to mark queens or nail polish of conspicuous colors are recommended. The colors and size of the marks used to identify individuals must be visible and readily identifiable at 1.5–2 m, which is a typical distance of an observer from a bait station. Ideal colors are those that contrast with the body parts of the hornets. Similar colors or color combinations on individuals visiting the same bait station or within the same foraging range are best avoided to prevent identities being confused. Individual identification is a crucial refinement in this method since, in areas of high hornet density, a single bait can attract hornets from multiple nests, making nest locating by triangulation of flying routes or by step-by-step deciphering of the foraging route, without distinguishing marks impossible. To further augment the delineation of the proximate nest location, we combine triangulation with nest distance, as estimated from the time the hornet spent flying to the nest and back to the bait. Thus, the procedure in this phase of the search is as follows.

**Figure 2:**
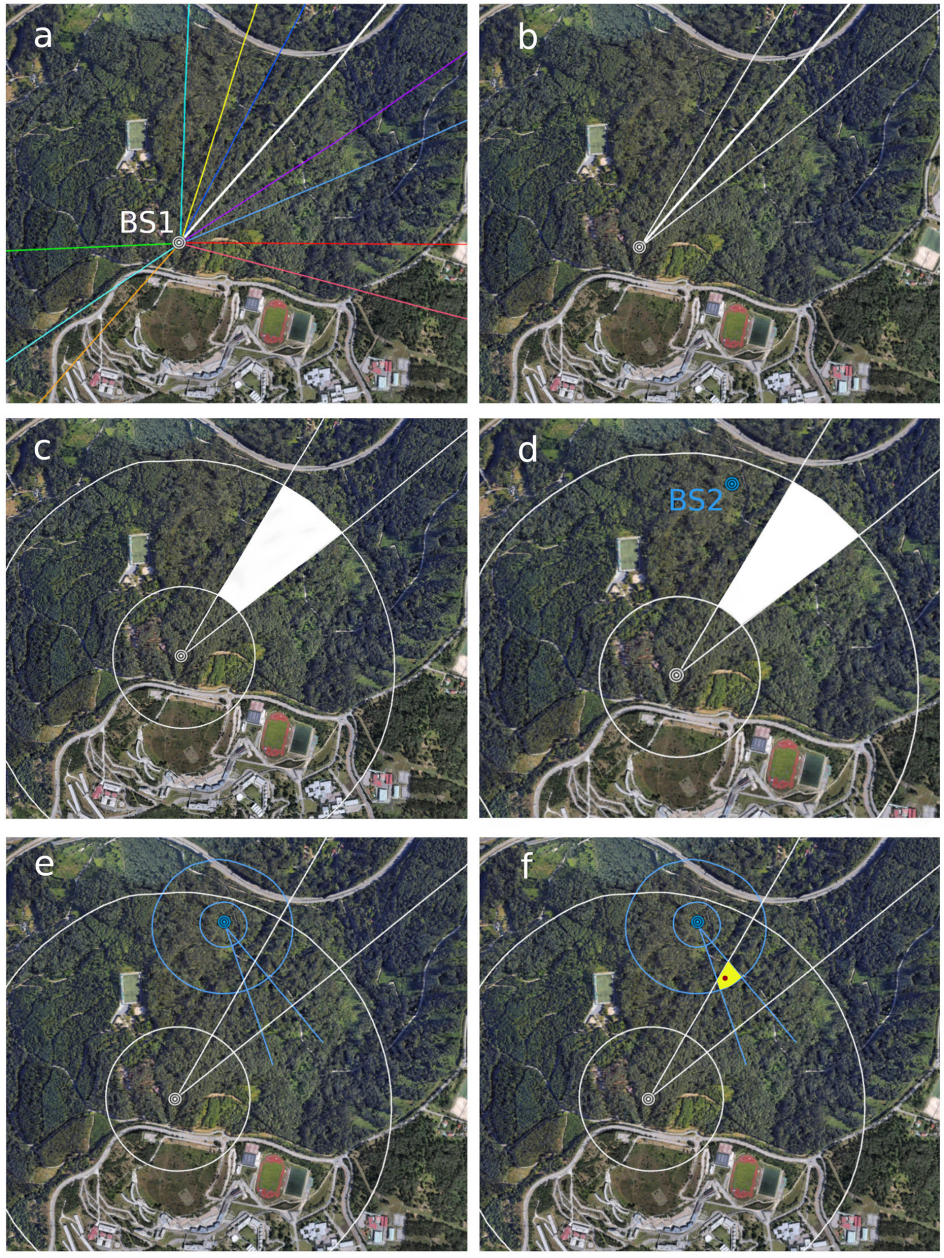
Tracing routes and distances to determine the likely area of nest location (first phase of the search). First, record and draw directions of several individuals from a protein bait station [BS1 in **(a)**]. Focus on the trajectory of one individual with consistent measurements of time and draw two external lines at 10 (for round-trip time < 10 min) or 15° (for round-trip time > 10 min) **(b)**. Then, draw concentric lines for D_min_ and D_max_ to find the likely location of the nest **(c)**. If this is too large, place a second bait station [BS2 in **(d)**] outside but near the first likely area and repeat the steps **(e)**. The convergent area is smaller [yellow area in **(f)**] increasing the chances of finding the nest after a visual search. The nest [red dot in **(f)**] was located at 22 m high in the tree top of a eucalypt.

Once released, marked specimens return to the bait, the direction taken from the bait station toward the nest and the bait-nest-bait round-trip time is recorded. The compass directions of several round trips of a number of individuals are traced on a map (Figure 2a). On obtaining several measurements for different individuals, attention is focused on the trajectory of one individual with consistent measurements of time (Figure 2b). Then, the mean vanishing direction for this individual, taken from a number of return-trips, is drawn on the map and used to define two external lines (Figure 2b). The external lines are drawn at 10° or 15° from the central line. The criteria to choose the angle is the minimum round trip time for each individual: 10° angle for times shorter than 10 min and 15° angle for times longer than 10 min. The reason for broadening the angle when time is >10 min is that, the larger the distance to the nest the higher the probability that the hornet deviates from the central vanishing direction observed, thus the likely area has to be enlarged.

After obtaining measurements of bait-nest-bait times for several round-trips (at least three trips), use the shortest time to estimate the maximum and minimum distances to the nest, we used the following formula:


$$\begin{array}{l} D_{\min}=\left(\;\frac{t_{t}-t_{n}}{2}\right)*S_{\min}\; \end{array}$$

$$\begin{array}{l} D_{\max}=\left(\;\frac{t_{t}-t_{n}}{2}\right)*S_{\max}\; \end{array}$$

where D_max_ and D_min_ are the maximum and minimum estimated distances, respectively; t_t_ is the round trip time, estimated as the minimum time recorded to complete the bait-nest-bait round trip by a given individual; t_n_ is a constant in the formulae corresponding to the estimated time spent on the nest (t_n_ = 45 s, as estimated in the field from a sample of 20 trips of different individuals); and S_min_ and S_max_ are the minimum and maximum flight speeds (S_min_ = 1.8 and S_max_ = 5.4 m/s).

The minimum flight speed was measured in the field from a sample of 30 observed flights (mean 1.8 m/s ± 0.32 SD; S. Rojas-Nossa unpubl. data) of individuals with different flight capacities and loads, and the maximum speed was obtained from the maximum speed reported for homing hornets measured with harmonic radar (12); our estimated maximum speed in the field was 2.7 m/s, but we use the maximum speed reported in the literature, for a more conservative estimate of the likely area of the nest, since this results in longer estimated distances and thus increases the search area for the nest location. These estimations are faster than those obtained by Sauvard et al. (14) in the lab and similar to those estimated by Maggiora et al. (11) in the field. Rainy weather or strong wind can modify the flight speed of the hornets (Rojas-Nossa Pers. Obs.), so it is recommended avoiding taking measurements under such conditions.

The shortest and longest estimated distances are represented in the map as two concentric circles centered on the bait station (Figure 2c). Once the directions and distances are delineated, a visual analysis of the lines will help to detect the likely location of a nest (white area in Figure 2c). If the likely area is still large, the placement of a second protein bait station in the margin of this area is advisable in order to reduce it to a more manageable size (Figure 2d). We suggest placing this station near but outside the likely area because hornets tend not to visit protein baits in the immediate vicinity of the nest. In each case, the particular conditions of the terrain and the facility to observe the vanishing directions of homing hornets must be taken into account in the location of bait stations. After following the steps as described for the first bait station (Figure 2e), the analysis of the convergent areas is useful to delimitate a narrower likely area (yellow area in Figure 2f). A detailed visual inspection, with the help of binoculars, in the field of the resulting area of convergence in search for the nest is usually successful. But in case detection is not yet achieved, one would proceed with the second phase of the search.

In the second phase, the baits are placed in the margin of the proximate nest area as previously delineated but, instead of protein, one would use an attractant based on alcoholic drinks, such as wine or beer, and sugar (15). This can be enhanced with pieces of honey comb heated in water, for better propagation of the scent. The objective is to generate an abundant flow of individuals between the nest and the feeder. Sometimes marking specimens is not required, as the proximity of the nest makes the provenance point of a large number of hornets easily evident, pointing to its location. However, the use of conspicuous marks makes hornets more visible and facilitates their tracking. In this case, it is advisable to anesthetise the individuals to be marked, in order to avoid stings. We used CO_2_ or cold narcosis for anesthetizing the hornets, which are commonly used to manipulate hymenopterans (5).

The marks used at this stage should allow the visualization of individuals from some distance and among the vegetation. Strips of fabrics or ribbons of fluorescent pink or orange color, with an approximate size of 6.5 × 60 mm provided good results in our area, but the colors and sizes selected may change depending on the environment. A thread or dental floss is knotted through one of the ends of the strip to tie it around the hornet petiole. To immobilize the hornet, a plate as suggested by Kennedy et al. (5) is useful, but forceps or queen-marking plunge cages for beekeeping are also helpful. Once released, hornets often perch on the vegetation and spend some time trying to remove the marks, and only start flying consistently from the feeders to the nest once they are accustomed to the marks. To avoid this delay, bees or flies can be offered to the hornet before release, so that they fly directly to the nest, allowing for immediate tracking. Marked individuals can be both visually and physically followed along the route to their nest, by maintaining an eye on the flying insect while moving in the same direction, so far as safety of observer is not compromised by the terrain. An alternative is to divide tasks between observers, one watching and communicating the departure of individuals from the feeder to a colleague, while the colleague advances along the flight path, in a sort of relay race. This should reveal the exact location of the nest.

## Results

The advantage of this method is the low cost of equipment and the simplicity to operate it, in comparison with other tracking methods such as radio-tracking or harmonic radar (5, 12). The total time required to find nests can vary highly ranging from some hours to several days, depending on aspects, such as the distance between the first bait station and the nest, the ground conditions, the type and density of vegetation, and observer performance during the different tasks. The method can be performed by a single observer, but a team of at least two people is recommended. Due to strict regulations governing the intentional or accidental release of invasive alien species into the environment [e.g., Regulation (EU) 1,143/2,014 on invasive alien species], it is necessary to obtain permits of authorization for release of marked hornets.

## Discussion

The method described is a refinement of previous methods of visual tracking, devised to determine the precise location of nests in areas where *V. velutina* is well-established and the high density of nests impairs the ability to visually track hornets from bait stations, due to the abundance of individuals from different nests. This method has proven useful in highly diverse environments with dense vegetation and constitutes an inexpensive alternative that requires limited training, making it feasible to be adopted by a wide range of people, including beekeepers and volunteers. This is especially advantageous in infested areas, where exhaustive efforts in extensive areas are required to localize and destroy a significant number of nests, for effective control of the hornet invasion.

## Data Availability Statement

Publicly available datasets were analyzed in this study. This data can be found here: https://figshare.com/s/18b2f1cc9833b7c580d5 Figshare repository, doi: 10.6084/m9.figshare.17125517.

## Author Contributions

SR-N and PÁ conducted the field work and the data analysis. SR-N drafted the manuscript. All authors designed the method, contributed to the article, and approved the submitted version.

## Funding

This work was funded by the Diputación de Pontevedra through the cooperation agreement 2017CONVDEPOAVISPÓN-BA2. The Interreg Atlantic Area Program (European Regional Development Fund, European Union) supported part of the work of SR-N and JG through the Atlantic-POSitiVE project (EAPA_800/2018).

## Conflict of Interest

The authors declare that the research was conducted in the absence of any commercial or financial relationships that could be construed as a potential conflict of interest.

## Publisher's Note

All claims expressed in this article are solely those of the authors and do not necessarily represent those of their affiliated organizations, or those of the publisher, the editors and the reviewers. Any product that may be evaluated in this article, or claim that may be made by its manufacturer, is not guaranteed or endorsed by the publisher.
